# Coating carbon nanotubes with a polystyrene-based polymer protects against pulmonary toxicity

**DOI:** 10.1186/1743-8977-8-3

**Published:** 2011-01-21

**Authors:** Lyes Tabet, Cyrill Bussy, Ari Setyan, Angélique Simon-Deckers, Michel J Rossi, Jorge Boczkowski, Sophie Lanone

**Affiliations:** 1 INSERM U955, Créteil, F-94010 France, and Université Paris Est Val de Marne (UPEC), Créteil, F-94010, France; 2 Institut universitaire romand de Santé au Travail (Institute for Work and Health), Université de Lausanne et Université de Genève, Rue du Bugnon 21, CH-1011 Lausanne, Switzerland; 3 Laboratory of atmospheric chemistry (LAC), Paul Scherrer Institut (PSI), OFLA008, CH-5232 Villigen PSI, Switzerland; 4 AP-HP, Hôpital Henri Mondor, Service de Physiologie Explorations Fonctionnelles, 94010 Créteil, France; 5 Hôpital Intercommunal de Créteil, Service de pneumologie et pathologie professionnelle, Créteil, 94000, France; 6 Department of Environmental Toxicology, University of California, Davis, CA 95616, USA

## Abstract

**Background:**

carbon nanotubes (CNT) can have adverse effects on health. Therefore, minimizing the risk associated with CNT exposure is of crucial importance. The aim of this work was to evaluate if coating multi-walled CNT (MWCNT) with polymers could modify their toxicity, thus representing a useful strategy to decrease adverse health effects of CNT. We used industrially-produced MWCNT uncoated (NT1) or coated (50/50 wt%) with acid-based (NT2) or polystyrene-based (NT3) polymer, and exposed murine macrophages (RAW 264.7 cell line) or Balb/c mice by intratracheal administration. Biological experiments were performed both *in vitro *and *in vivo*, examining time- and dose-dependent effects of CNT, in terms of cytotoxicity, expression of genes and proteins related to oxidative stress, inflammation and tissue remodeling, cell and lung tissue morphology (optical and transmission electron microscopy), and bronchoalveolar lavage fluid content analysis.

**Results:**

extensive physico-chemical characterization of MWCNT was performed, and showed, although similar dimensions for the 3 MWCNT, a much smaller specific surface area for NT2 and NT3 as compared to NT1 (54.1, 34 and 227.54 m^2^/g respectively), along with different surface characteristics. MWCNT-induced cytotoxicity, oxidative stress, and inflammation were increased by acid-based and decreased by polystyrene-based polymer coating both *in vitro *in murine macrophages and *in vivo *in lung of mice monitored for 6 months.

**Conclusions:**

these results demonstrate that coating CNT with polymers, without affecting their intrinsic structure, may constitute a useful strategy for decreasing CNT toxicity, and may hold promise for improving occupational safety and that of general the user.

## Background

Carbon nanotubes (CNT) exhibit unique properties, including mechanical, thermal and electrical conductivity, as well as field emission properties. These properties are associated with many applications (car industry, sport accessories, ...), and lead to a steady increase in the industrial production of CNT. However, it is increasingly obvious that exposure to nanoparticles in general, and CNT in particular, can have adverse effects on human health, especially at the level of the pulmonary system, which is a primary route of exposure [1]. This is raising considerable concern [2-9]. Therefore, minimizing the risk associated with CNT exposure is of crucial importance.

Among adverse health effects secondary to exposure to CNT, inflammation and oxidative stress are particularly worrisome because they can be associated with tissue remodeling and impaired function and/or carcinogenesis [10]. Recent data show that the length and rigidity of the nanotubes influences the pro-inflammatory effect of CNT [11]. However, the possible influence of other physicochemical properties remains incompletely understood [12]. Since surface characteristics influence the pro-inflammatory effect of spherical nanoparticles [13], we hypothesized that embedding CNT in polymers, thus modifying the surface environment of those CNT, could modify their toxicity, and thus represent a useful strategy to decrease adverse health effects of industrially-produced CNT, without affecting their specific properties and further applications. The protective effect of the compatible solute Ectoine against carbon nanoparticle-induced lung inflammation has been nicely demonstrated recently [14]. However, a strategy based on the production of intrinsically safer nanomaterials seems to be much more promising. Such an approach is essential to the further development and safe use of CNT, at an occupational level as well as at the level of the general user.

To assess our hypothesis, we utilized an original approach by coating industrially produced multi-walled CNT (MWCNT) with two different polymers (acid-based and polystyrene-based, respectively) that resulted in different surface environment but similar specific surface areas. To the best of our knowledge, although surface modifications is important to CNT toxicity [15-19], polymer coating has never been used before as an approach to modulate CNT toxicity. Coating could represent a useful tool, as it allows modifying CNT's external surface environment, without affecting their intrinsic structure. We investigated whether coating modified the toxicity of MWCNT *in vitro *in murine macrophages and *in vivo *in mice monitored for 6 months after intratracheal MWCNT instillation. Here, we report that MWCNT-induced cytotoxicity, oxidative stress, and inflammation in both models were increased by acid-based polymer coating and decreased by polystyrene polymer coating. These results demonstrate that surface characteristics play a major role in the biological response to CNT and that modifications of the surface environment by coating with specific compounds may constitute a useful strategy for decreasing CNT toxicity, without affecting their intrinsic structure, already at the time of their production. This may therefore hold promise for improving occupational safety as well as that of general users.

## Methods

We investigated the effects of industrially-produced MWCNT that were either uncoated (NT1) or coated with carboxylic polyacid polymer (NT2) or a hydrophobic polystyrene polybutadiene polymethylmethacrylate polymer (NT3). These MWCNT were provided by ARKEMA-France (Colombes, France). The two coated MWCNT were composed of 50% MWCNT and 50% coating polymer by weight (determined by Thermo Gravimetric Analysis). Coatings were performed as follows; both coated MWCNT were produced by impregnation of NT1 powder (50% weight) with a solution of polymer (carboxylic polyacid polymer for NT2, and polystyren polybutadiene polymethylmethacrylate for NT3), the solvent being thereafter eliminated by drying under vacuum conditions. Solvent was water for NT2, and methyl ethyl cetone for NT3. NT2 remained coated after dispersion in the culture medium, as revealed by the Toluidine Blue (TB) assay, which measures the stability of polymer grafting [20] (data not shown). Briefly, a 5.10^-4 ^M TB aqueous solution was prepared and 0.2 ml of a buffer solution of 2-amino 2-methyl propanol was added to reach and maintain the solution pH value at 10. Each MWCNT sample was placed in TB solution at 30°C up to 24 hours. At different time points, uncomplexed TB molecules were removed by rapid washing (30 minutes) of the sample by a basic aqueous solution (NaOH 5.10^-4 ^M, pH 9). Then, each sample was in 10 ml of an aqueous acetic acid solution (50% v/v) for 24 hours to obtain complete decomplexation of TB from the sample. The decomplexation solution was analyzed by UV/visible spectroscopy at 633 nm. There was no chemical link between the polymers and the nanotubes.

Both coated NT (NT2 and NT3) appear as powders, as the pristine one (NT1), and were dispersed in culture medium for both *in vitro *and *in vivo *experiments. The two polymers were also dispersed in culture medium in order to assess their intrinsic effects.

The effects of the three MWCNT were compared to those of crocidolite asbestos fibres (80 nm in diameter) kindly donated by Dr. Ghislaine Lacroix (INERIS, Verneuil-en-Hallatte-France) and of nanosized carbon black (CB, FR103, 95 nm in diameter; Degussa, Dusseldorf, Germany).

We chose to study industrially-produced MWCNT to obtain results relevant to occupational health, in the context of increasing industrial development and use of CNT.

### Physicochemical characterization of multi-walled carbon nanotubes

MWCNT dimensions were measured using transmission electron microscopy (TEM), and MWCNT morphology was observed using scanning electronic microscopy (SEM). Chemical composition and carbon content were determined using inductively coupled plasma mass spectroscopy (ICP-MS), electron spectroscopy for chemical analysis (ESCA). Specific surface area was measured using Brunauer Emmett Teller (BET) adsorption isotherms of nitrogen at 77 K. Surface functional groups were identified by surface titration using six probe gases flowing across a Knudsen flow reactor [21-23]. The relative uncertainty for both values (substrate/probe gas pair) is typically 25%.

### Particle suspensions

All particles were suspended at 10 mg/ml in Dulbecco's modified Eagle medium (DMEM). We choose not to disperse CNT using any particular agent because our aim was to examine the biological effects of surface properties of coated and uncoated CNT without any other interference. The suspensions were then vortexed for 1 min, and sonicated (Elma S30H, 50-60 Hz) for 30 min under cooling conditions, with a 30-s interruption every 10 min with the vortex at maximum speed. Immediately after the end of sonication, the particle solutions were vortexed again for 1 min at maximum speed and diluted to various concentrations in culture medium (see below). CNT dispersion was quantified by dynamic light scattering using a Zetasizer Nano S (Malvern Instruments Ltd, Worcestershire, UK). Under control conditions, cells were cultured in serum-free DMEM without the particles (see below).

### In Vitro Studies

#### a. RAW 264.7 macrophage culture and exposure

RAW 264.7 murine macrophages were purchased from the American Type Culture Collection (Manassas, VA), and were cultured as previously described [24]. Cells were exposed for 6 or 24 hours to 0.1 to 100 μg/ml (0.02-20 μg/cm^2^) of MWCNT or to 100 μg/ml (20 μg/cm^2^) of crocidolite fibres or CB nanoparticles in serum-free medium, as it is known that serum can interact with nanomaterials and modify cellular response [25].

#### b. Light microscopy

Cell morphology was assessed by light microscopy after cell staining with Harris haematoxylin-phloxin. The size and number of the agglomerates present on the cells were determined as previously described [23]. Light microscopy studies were done in blinded fashion by two independent observers (LT and CB). The coefficient of variation for each measurement was <5%.

#### c. Cell viability

Cell viability was assessed using three methods, namely the MTT and Neutral Red assays and quantification of DNA content. These tests were performed as previously described [23]. Results were expressed as the mean of at least three independent experiments, each having six replicates, given as the ratio of the mean under each condition over the mean under the control condition (cells exposed to DMEM). Since nanomaterials may interfere with cytotoxicity tests [26], we performed the assays with and without 100 μg/ml of NT1, NT2, or NT3 during incubation with the dye, and we measured absorbance. No interference of NT1, NT2, or NT3 with any of the assays was observed (data not shown).

#### d. Transmission Electron Microscopy

Cells exposed for 24 h to 10 μg/ml of NT1, NT2, or NT3 were examined by TEM as previously described [23]. The percentage of cells with MWCNT-containing vacuoles after 24 hours' exposure to 100 μg/ml was determined on semi-fine slides. Under each stimulation condition, five fields were randomly selected from top to bottom across the vertical diameter of the sample. The measurements were made in blinded fashion by two independent observers (LT and CB). The coefficient of variation for the measurement was <5%.

#### e. Reverse Transcription and Quantitative PCR (Q-PCR)

The mRNA expression of various genes involved in oxidative stress and inflammation was measured using Q-PCR as described previously [23]. The primer sets are shown in Table 1. The expression of the gene of interest was reported as the ratio over RpL13 expression.

**Table 1:** Sequences of primers used for quantitative PCR assays

Gene	Forward Primer	Reverse Primer
*RPL-13*	GTGGTCCCTGCTGCTCTCCAA	CGATAGTGCATCTTGGCCTTTT
*HO-1*	CACGCATATACCCGCTACCT	CCAGAGTGTTCATTCGAGCA
*GPX-1*	TGAAGAGATTCTGAATTCCCTCAAG	CAGGAAGGTAAAGAGCGGGTG
*SOD-1*	CAAATTACAGGATTAACTGAAGGCC	GGCCACCATGTTTCTTAGAGTGAG
*SOD-2*	CTACGTGAACAATCTCAACGCC	ATTAATATGTCCCCCACCATTGAAC
*TNF-α*	CTGTCTACTGAACTTCGGGGTGAT	GGTCTGGGCCATAGAACTGATG
*CXCL2*	GAACATCCAGAGCTTGAGTGTGAC	CTTGCCTTTGTTCAGTATCTTTTGG
*α-2 collagen-1*	GGCTATGACTTTGGTTTTGAAGGA	CGTTGTCGTAGCAGGGTTCTTT
*α-1 collagen-3*	CCAGAACATTACATACCACTGCAAA	GTGTTTAGTACAGCCATCCTCTAGAACTG

*RPL13*, ribosomal protein L13; *HO-1*, heme oxygenase-1; *SOD*, superoxide dismutase; *GPX-1*, Glutathione peroxidase-1; *CXCL2*, macrophage inflammatory protein-2; *TNF-α*, tumour necrosis factor alpha.

### In Vivo Studies

#### a. Animal exposure

The experiments were approved by the local Institutional Animal Care and Use Committee, and the experimental protocol complied with French legislation about animal studies. Male Balb/C mice aged 7-9 weeks and weighing 22 ± 0.23 g, were purchased from Janvier (Le Genest-St-Isle, France) and housed in standard wire-topped cages in temperature-controlled units with food and water ad libitum.

NT1, NT2, or NT3 were suspended in DMEM as for the *in vitro *studies. The suspension was instilled intratracheally after anaesthesia of the animal with 1.6 mg ketamine (Merial, Lyon, France) plus 300 mg xylazine (Bayer, Puteaux, France). A single dose (10 or 100 μg/mouse) of NT1, NT2, or NT3 was given to each animal. The highest dose was relevant to the 20 μg/cm^2 ^concentration used in the *in vitro *studies [27]. The mice were sacrificed 1, 7, 30, 90, or 180 days post-instillation. In a subset of experiments, animals were exposed to 200 μg of NT2 or NT3, in order to allow adequate comparison with 100 μg NT1.

#### b. Bronchoalveolar Lavage (BAL) and Lung Recovery

The mice were anesthetized with an intramuscular injection of ketamine/xylazine and sacrificed by exsanguination. BAL fluid analysis and lung tissue recovery were performed as described previously [28]. The percentage of macrophages with MWCNT-containing vacuoles was determined as for the *in vitro *studies. The mRNA expression of the genes evaluated *in vitro *was measured using Q-PCR on homogenates, with the same set of primers (Table 1). In addition, we measured the expression of collagen-1 and -3, used as markers for interstitial fibrosis.

#### c. Histological study of lung samples

Lung histology was examined in a subset of animals different from that used for investigating BAL fluid and lung gene expression determination. The lungs were fixed with 0.8 ml of a 1:1 mix of 0.9% saline with Tissue-Teck ornithine carbamoyltransferase fluid (Sakura, Zoetervoude, The Netherlands) and were snapfrozen in liquid nitrogen. Lung histology studies were performed as described previously [29].

The number and size of MWCNT agglomerates in the lungs of animals exposed for 24 hours, 1 month, or 6 months was measured in five representative animals per group. For each animal, five fields were randomly selected from top to bottom across the vertical diameter of the section. Calculations were performed using ImageJ software (http://rsbweb.nih.gov/ij/), and the analysis was performed in blinded fashion by two independent observers (LT and CB). The coefficient of variation for each measurement was <5%.

### Statistical Analysis

The values from at least four different experiments (*in vitro *study) and 6-8 animals (*in vivo *study) are represented as box and whisker plots. *In vitro *experiments were performed in triplicate. The data were analysed using the non-parametric Kruskal-Wallis test, followed by Dunn's multiple comparison test where appropriate. For all tests, *p *values smaller than 0.05 were considered significant.

## Results

### Physicochemical characterization of multi-walled carbon nanotubes

The NT1 used in this study have been extensively characterized [23]. NT2 and NT3, the two coated MWCNT were composed of 50% MWCNT and 50% coating polymer by weight. They were produced by chemical vapour deposition on a supported catalyst in a fluidized bed that yielded spheric heaps of MWCNT entangled around the supported catalyst, measuring about few hundred microns in diameter, and forming a free-flowing powder.

**Figure 1:**
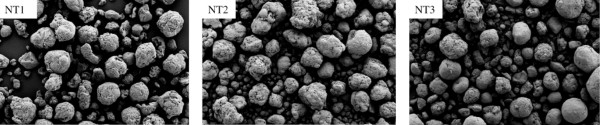
**SEM observations**. Representative scanning electron microscopy (SEM) image of uncoated multi-walled carbon nanotubes (NT1) and of the same nanotubes coated with carboxylic polyacid polymer (NT2) or polystyrene polybutadiene polymethacrylate polymer (NT3).

Typical SEM images of the various MWCNT are shown in Figure 1 and Additional file 1, figure S1. Physicochemical characterization (Table 2) showed that NT2 and NT3 had similar dimensions and metallic impurities compared to NT1. Specific surface area was similar for NT2 and NT3 and smaller than the specific surface area of NT1. Furthermore, all three MWCNT showed micrometric agglomerates in suspension (as demonstrated previously [23]), whose diameter was largest for NT1 and smallest for NT2. Surface chemical analysis of functional groups located on the surface of the MWCNT showed a larger amount of acidic sites on NT2 compared to the other two MWCNT (Table 3). Moreover both NT2 and NT3 contain a significant amount of surface carbonyl. No basic oxides are present, as seen by the ratio of the uptake of HCl/CF_3_COOH.

**Table 2:** Physicochemical characteristics of the multi-walled carbon nanotubes (MWCNT) used in the study

		NT1	NT2	NT3
**Coating polymer (50/50 wt%)**		**None**	**Carboxylic polyacid polymer**	**PMMA^*a *^polymer**

**Diameter (nm)**		12 ± 1	12 ± 1	12 ± 1

	0-15 nm	85%	85%	85%
	
**Size distribution (diameter)**	15-30 nm	13%	13%	13%
	
	>30 nm	2%	2%	2%

**Length (μm)**		0.1-13	0.1-13	0.1-13

**Metallic impurities**	Aluminium (%)	3.2	1.15	1.25
	
	Iron (%)	2.45	0.75	0.85

**Specific surface (m**^**2**^**/g)**		227.4	54.1	34

**Agglomerate size - mean diameter (%<10 μm)**		338 (0.13)	118 (1.45)	267 (0.83)

^*a *^Polystyrene polybutadiene polymethylacrylate (PMMA).

**Table 3:** Characterization using a Knudsen flow reactor of the surface functional groups present on the multi-walled carbon nanotubes (MWCNT)

MWCNT	Gas [gas-phase probe molecules]	**N(CH**_**3**_**)_3_** [acidic sites]	HCl [basic sites]	**CF**_**3**_**COOH** [basic sites]	**NH**_**2**_**OH** [carbonyl functions]	O_3_ [oxidisable sites]	NO_2_ [oxidisable sites]
**NT1**	**N°/mg**^***a***^	3.0 · 10^15^	8.1 · 10^16^	2.4 · 10^16^	2.4 · 10^17^	3.9 · 10^18^	1.2 · 10^16^
	
	**N°/cm**^**2**^^***b***^	1.32 · 10^12^	35.6 · 10^12^	10.6 · 10^12^	105.5 · 10^12^	1715 · 10^12^	5.3 · 10^12^

**NT2**	**N°/mg**^***a***^	6.4 · 10^15^	4.4 · 10^16^	5.9 · 10^15^	3.4 · 10^17^	1.8 · 10^17^	1.2 · 10^15^
	
	**N°/cm**^**2**^^***b***^	11.8 · 10^12^	81.3 · 10^12^	1.1 · 10^12^	628.5 · 10^12^	332.7 · 10^12^	2.2 · 10^12^

**NT3**	**N°/mg**^***a***^	2.6 · 10^14^	1.4 · 10^16^	5.8 · 10^14^	2.0 · 10^17^	1.1 · 10^18^	1.9 · 10^15^
	
	**N°/cm**^**2**^^***b***^	0.8 · 10^12^	41.2 · 10^12^	1.7 · 10^12^	588 · 10^12^	3235 · 10^12^	5.6 · 10^12^

^*a *^Number of probe molecules taken up per mg of deposited nanoparticles.

^*b *^Number of probe molecules taken up per square cm of MWCNT (no/cm^2^), using the BET surface as a surface metric.

NT1, uncoated MWCNT; NT2, MWCNT coated with carboxylic polyacid polymer; NT3, MWCNT coated with polystyrene polybutadiene polymethylacrylate polymer.

### In vitro studies

**Figure 2:**
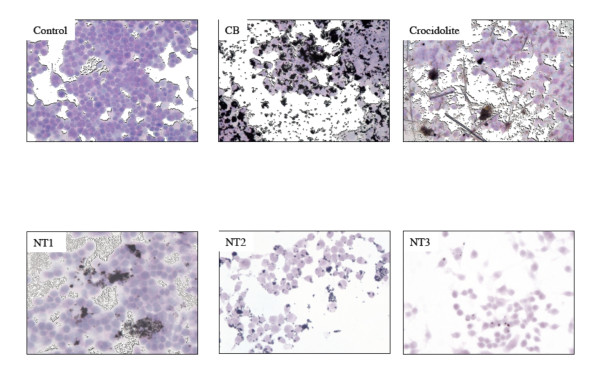
**Effects of MWCNT on macrophages in vitro - light microscopy observations**. Representative light microscopy images of RAW 264.7 cells exposed for 24 h to culture medium alone (Control) or 100 μg/ml of carbon black (CB), crocidolite fibres, NT1, NT2 or NT3. Original magnification: x 20. Abbreviations are the same as in Figure 1.

**Figure 3:**
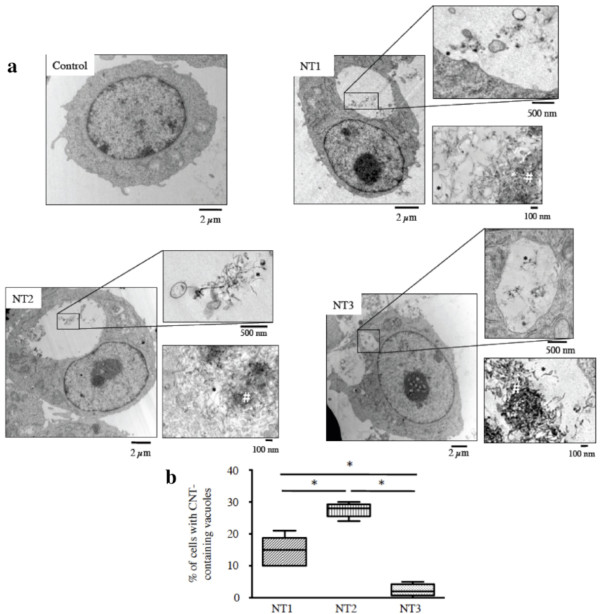
**Effects of MWCNT on macrophages in vitro - TEM observations**. **Panel a**: Representative transmission electron microscopy (TEM) images of RAW 264.7 cells exposed for 24 h to culture medium alone (Control), or 10 μg/ml of NT1, NT2, or NT3. Inserts show details. *: example of individual carbon nanotubes. #: example of carbon nanotube agglomerates. Abbreviations are the same as in Figure 1. **Panel b**: Quantification of cells with carbon nanotube-containing vacuoles, expressed as the percentage of the total number of cells. Results are represented as box and whisker plots of values from five fields. Abbreviations are the same as in Figure 1, *: *p *< 0.05.

We first evaluated the effects of the three MWCNT on macrophages (murine cell line RAW 264.7), which play a key role in host defence mechanisms against foreign bodies. Figure 2 shows representative light microscopy images of RAW macrophages exposed for 24 h to the three MWCNT, CB, and asbestos fibres. Even after thorough washing, MWCNT agglomerates were seen in contact with the cells. The number of these agglomerates was largest for NT2 and smallest for NT3 (Table 4). All three MWCNT were internalized by the macrophages within vacuoles, as single nanotubes or as agglomerates, without any modification of cell morphology (TEM images, Figure 3a and Additional file 2, figure S2). Quantitative analysis of internalization in vacuoles showed that the percentage of cells containing internalized nanotubes was greatest with NT2 and smallest with NT3 (Figure 3b). Few MWCNT were seen in the cytoplasm. Taken together, these results indicate stronger "attachment" to the cell surface and greater internalisation with NT2 than with NT1 or NT3, despite the similar dimensions, metallic impurities of the three MWCNT, and a specific surface similar to NT3.

**Table 4:** Size and number of carbon nanotube agglomerates found in vitro 24 hours after exposure to multi-walled carbon nanotubes (MWCNT)

MWCNT	**Surface area **(μm^2^)	Number/field
**NT1**	30.23 ± 7.32	18.5 ± 1.5

**NT2**	12.87 ± 3.4*	37.3 ± 3.7*

**NT3**	4.97 ± 1.61^#^	9.3 ± 1.9^#^

*: *p *< 0.05 vs. NT1.

#: p < 0.05 vs. NT1 and NT2.

NT1, uncoated MWCNT; NT2, MWCNT coated with carboxylic polyacid polymer; NT3, MWCNT coated with polystyrene polybutadiene polymethylacrylate polymer.

**Figure 4:**
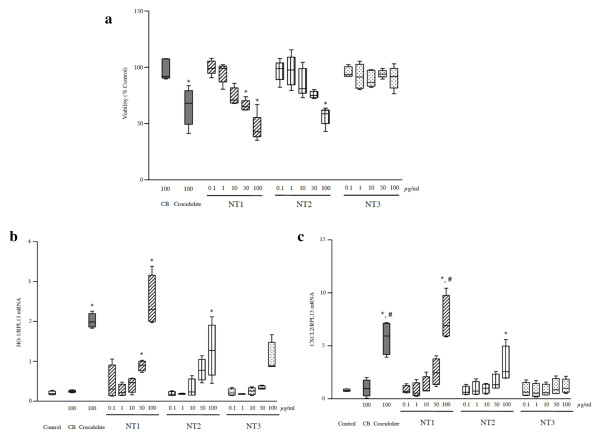
**Effects of MWCNT on macrophages in vitro - viability and mRNA content**. **Panel a**: Cell viability assessed using the MTT assay. Viability was expressed as the percentage of control cell values after 24 h exposure to 100 μg/ml CB, crocidolite, or NT1, NT2, and NT3 in doses of 0.1 to 100 μg/ml. Abbreviations are the same as in Figures 1 and 2. Results are represented as box and whisker plots of values from 3-6 experiments. *: *p *< 0.05 *vs*. control condition. **Panel b**: *HO-1 *mRNA expression in RAW 264.7 cells after 6 h exposure to culture medium alone, 100 μg/ml CB, crocidolite, or NT1, NT2, and NT3 in doses of 0.1 to 100 μg/ml. Results are expressed as the ratios over RPL13 mRNA levels and represented as box and whisker plots of values from 3-6 experiments. Abbreviations are the same as in Figures 1 and 2, *: *p *< 0.05 *vs*. control condition. **Panel c**: *CXCL2 *mRNA expression in RAW 264.7 cells after 24 h exposure to culture medium alone, 100 μg/ml CB, crocidolite, or NT1, NT2, and NT3 in doses of 0.1 to 100 μg/ml. Results are expressed as ratios over RPL13 mRNA levels and represented as box and whisker plots of values from 3-6 experiments. Abbreviations are the same as in Figures 1 and 2, *: *p *< 0.05 *vs*. control condition, #: *p *< 0.05 *vs*. NT2.

Cell viability was assessed using several tests. The MTT assay showed a similar dose-dependent decrease in mitochondrial metabolism after 24 h of exposure to NT1, NT2, and crocidolite (Figure 4a). This effect was already detectable after 6 h with NT2 (Additional file 3, figure S3). No such effect was observed with NT3, CB, or the polymers alone (data not shown). The Neutral Red assay (which measures cell membrane permeability) and DNA content quantification (which measures cell number) showed no significant alterations in cells exposed to any of the MWCNT or other materials (data not shown). The preferential effect of nanoparticles on mitochondrial metabolism has been described previously [23,30-32].

We next quantified the expression of several genes involved in inflammation and oxidative stress. Expression of the mRNAs for the antioxidant gene *HO-1 *and the pro-inflammatory gene *CXCL2 *was significantly increased in cells exposed to crocidolite, NT1, or NT2, compared to control cells exposed to medium alone (Figures 4b and 4c). NT1 induced a significantly greater increase in *CXCL2 *expression than did NT2 (*p *< 0.05, Figure 4c). No such modifications occurred after cell incubation with NT3, CB or the polymers alone (data not shown). Finally, with none of the nanomaterials studied were changes seen in mRNA expression of the antioxidant genes *GPX-1*, *SOD-1*, or *SOD-2*, or of the inflammatory gene *TNF-a *(data not shown).

### In vivo studies

**Figure 5:**
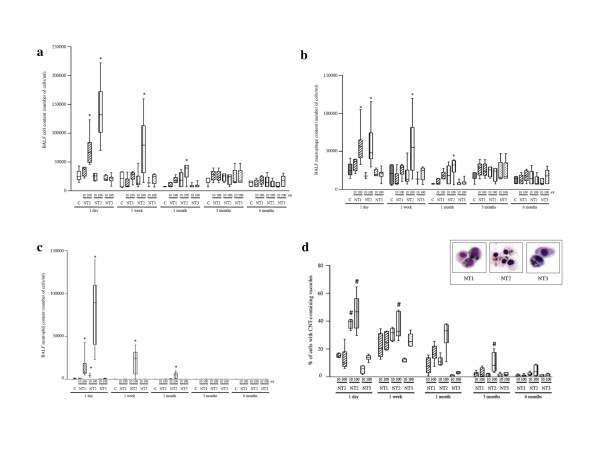
**Effects of MWCNT in vivo in mice - BALF analysis**. **Panel a**: Effect of NT1, NT2 or NT3 in a dose of 10 or 100 μg/mouse on total cell content of bronchoalveolar lavage fluid (BALF) after 1 to 180 days' exposure. Results are represented as box and whisker plots of values from 4-6 animals per group. Abbreviations are the same as in Figures 1 and 2. *: *p *< 0.05 compared to control mice. **Panel b**: Effect of NT1, NT2, or NT3 (10 or 100 μg/mouse) on total BALF macrophage content after 1 to 180 days' exposure. Results are represented as box and whisker plots of values from 4-6 animals per group. Abbreviations are the same as in Figures 1 and 2. *: *p *< 0.05 compared to control mice. **Panel c**: Effect of NT1, NT2, or NT3 (10 or 100 μg/mouse) on total BAL fluid neutrophil content after 1 to 180 days' exposure. Results are represented as box and whisker plots of values from 4-6 animals per group. Abbreviations are the same as in Figures 1 and 2. *: *p *< 0.05 compared to control mice. **Panel d**: Quantification of BAL macrophages with internalized carbon nanotubes, expressed as the percentage of the total macrophage number. Results are represented as box and whisker plots of values obtained from 4-6 animals per group. #: *p *< 0.05 *vs*. NT1 and NT3. Insert: representative light microscopy images of BAL cells after 1 day's exposure to 100 μg/mouse of NT1, NT2, or NT3 (magnification ×40). Abbreviations are the same as in Figure 1.

The relevance of the *in vitro *data was evaluated *in vivo *in Balb/c mice, each given a single intratracheal instillation of either 10 μg or 100 μg of MWCNT then monitored for up to 6 months. BAL fluid analysis showed that exposure to NT2 induced a dose-dependent increase in total cell count (*p *< 0.05 vs. control, Figure 5a) and a significant influx of neutrophils and macrophages (*p *< 0.05 vs. control, Figure 5b and 5c). These effects were observed 24 h post-instillation and lasted up to 1 month. With NT1, the modifications were similar to those produced by NT2 but less marked and present only 24 h post-instillation. No significant effect was observed after administration of polymers alone (data not shown). All three MWCNT were internalized in macrophages between 1 day and 1 month after instillation (Additional file 4, figure S4). The percentage of macrophages with MWCNT-containing vacuoles was higher with NT2 than with NT1 or NT3, and was also observed between 1 day and 1 month after instillation (*p *< 0.05, Figure 5d). MWCNT internalization was independent from the MWCNT dose. The percentages of macrophages with MWCNT-containing vacuoles were not significantly different between NT1 and NT3.

**Figure 6:**
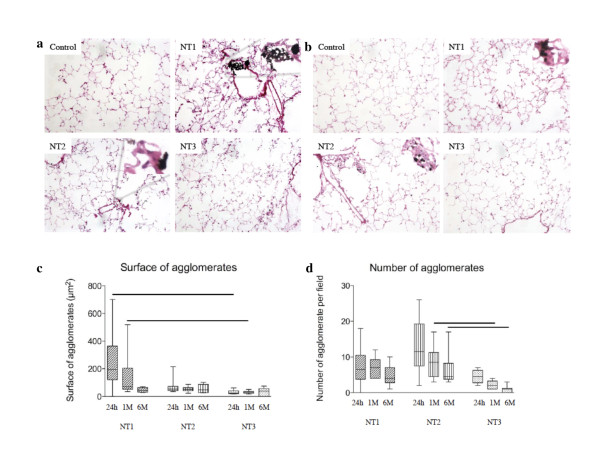
**Effects of MWCNT in vivo in mice - lung histology**. **Panel a**: Lung histology 1 day after a single intratracheal instillation of vehicle or NT1, NT2 or NT3 (100 μg/mouse, magnification ×20). Abbreviations are the same as in Figure 1. Inserts are higher magnification views (×40) of the carbon nanotube agglomerates. **Panel b**: Lung histology 30 days after a single intratracheal instillation of vehicle, NT1, NT2, or NT3 (100 μg/mouse, magnification ×20). Abbreviations are the same as in Figure 1. Inserts are higher magnification views (×40) of carbon nanotube agglomerates. **Panel c**: Quantification of the number and size of carbon nanotube agglomerates in lungs of mice 24 hours, 1 month, or 6 months after a single intratracheal instillation of 100 μg NT1, NT2, or NT3. Results are represented as box and whisker plots of values from 5 animals (five fields per animal). *: *p *< 0.05 between groups.

Histological studies of the lungs 24 h after instillation showed the presence of widespread micrometric MWCNT agglomerates, which were mainly located in the bronchiolar lumen and alveolar ducts with NT1 and near the bronchioles and in the alveoli with NT2. NT3 agglomerates were scarce and confined to the alveoli (Figure 6a and 6b). Agglomerate size was greatest with NT1 and smallest with NT3, whereas agglomerate number was greatest with NT2 and smallest with NT3 (Figure 6c). After 1 week, clusters of cells surrounding visible MWCNT agglomerates were seen in the lungs of animals exposed to NT1 or NT2. These clusters were mainly located near the terminal bronchioles but were also found in the alveolar ducts and alveoli. They were still present 6 months post-exposure (Additional file 5, 6, 7, 8, 9, and 10 figures S5, S6, S7, S8, S9, and S10). No evidence of fibrosis was found with any of the three MWCNT.

**Figure 7:**
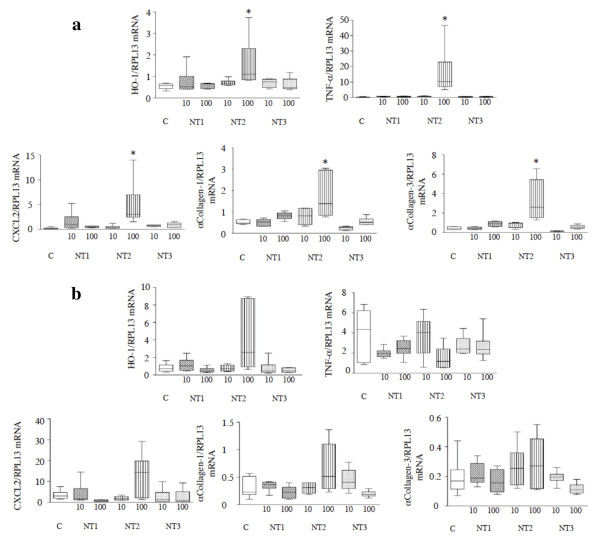
**Effects of MWCNT in vivo in mice - lung mRNA content**. **Panel a**: *HO-1*, *TNF-a*, *CXCL2*, *a-collagen-1*, and *a-collagen-3 *mRNA expression in the lungs of mice 7 days after intratracheal instillation of vehicle or of 10 or 100 μg of NT1, NT2, or NT3. Results are expressed as the ratios over RPL13 mRNA levels and represented as box and whisker plots of values from 5-6 animals. Abbreviations are the same as in Figure 1. *: *p *< 0.05 *vs*. control condition. **Panel b**: *HO-1*, *TNF-a*, *CXCL2*, *a-collagen-1*, and *a-collagen-3 *mRNA expression in the lungs of mice 180 days after intratracheal instillation of vehicle or of 10 or 100 μg of NT1, NT2, or NT3. Results are expressed as the ratios over RPL13 mRNA levels and represented as box and whisker plots of values from 5-6 animals. Abbreviations are the same as in Figure 1.

The mRNA expression of various genes implicated in oxidative stress, inflammation, and fibrosis was quantified in lung homogenates, as for the *in vitro *experiments. Significant increases in lung mRNA expression of *SOD-2 *and *HO-1*, *TNF-α *and *CXCL2*, and *collagen-1 *and *-3 *were observed between 1 day and 1 week post-instillation in animals exposed to NT2, compared to control animals (*p *< 0.05 for all comparisons, Figure 7d and data not shown). The expression of these genes returned to basal levels by 1 month post-instillation, except for *HO-1 *whose expression returned to basal levels only after 3 months (see Figure 7e for values at 6 months). No modifications in gene expression were seen in animals exposed to NT1, NT3 or polymers alone (data not shown). With none of the three MWCNT did changes occur in the expression of *GPX-1*, *SOD-1*, or *TGF-b *mRNA (data not shown).

## Discussion

Taken together, these results show that coating MWCNT with an acidic polymer reduced their specific surface five-fold but enhanced their dispersion in culture medium and their internalization by macrophages both *in vitro *and *in vivo*, compared to uncoated MWCNT. These results agree with earlier data [33] showing that the presence of acidic groups such as phenolic or carboxyl groups on the surface of carbon materials facilitates the internalization of these materials by mouse macrophages. The marked internalization of NT2 by mouse macrophages in our study was associated with the induction of a cytotoxic, oxidative, and inflammatory response despite the marked decrease in specific surface, compared to NT1. A similar direct relationship between the degree of internalization and the severity of the inflammatory response was described with metallic nanoparticles [34]. In contrast to the *in vitro *experiments, in which the oxidative and inflammatory effects of NT1 and NT2 were similar, the *in vivo *experiments showed stronger oxidative and inflammatory responses to NT2 than to NT1, suggesting that cells other than macrophages were involved in the response to NT2 *in vivo*. This hypothesis is supported by the smaller size and larger number of NT2 agglomerates in the alveolar wall, suggesting enhanced contact of alveolar cells with NT2 compared to NT1. Accordingly, Shvedova and coworkers [35] demonstrated that exposure of mice to relatively well-dispersed single-walled CNT by inhalation produced a stronger inflammatory response than did pharyngeal aspiration of an agglomerated particle suspension (micrometer-size agglomerates). A stronger inflammatory response to NT2 could result from their acidic surface, in addition to their better internalization. Indeed, we recently obtained data showing that the inflammatory response of murine macrophages exposed to MWCNT generated by catalyst chemical vapor deposition was more marked when acidic groups were present on the MWCNT surface, compared to identically produced MWCNT of similar length that did not have acidic groups on their surface (Cyrill Bussy, unpublished data). Moreover, Hamilton and colleagues [36] recently showed that titanium dioxide nanoparticles induced inflammasome activation and inflammatory cytokine release through a cathepsin B-mediated mechanism involving lysosomal disruption and, therefore, acidification of the intracellular compartment, thus linking acidification to inflammatory response. It should be noted, however, that the inflammatory response to NT2, evaluated based on BAL fluid cellularity and lung gene expression, resolved between 1 week and 1 month post-instillation, which probably explained the absence of persistent histological abnormalities. The absence of fibrosis may also reflect the strain of mice used in this study as compared to others [37].

In contrast to the acidic polymer, the polystyrene polymer suppressed the cytotoxicity, oxidative stress, and inflammation observed both *in vitro *and *in vivo *when the nanotubes were not coated. Several mechanisms may explain this result. First, the coated MWCNT contained 50% polymer by weight and therefore contained half the amount of nanotube material supplied by NT1. To investigate the potential role for this difference, we performed *in vivo *experiments, comparing the effects of 100 μg of NT1 to those of a "double dose" of NT2 and NT3 (200 μg, thus containing the same amount of MWCNT than 100 μg of NT1). These experiments showed that BAL fluid cellularity 24 h after exposure to 200 μg NT2 or NT3 was similar to that seen with the original dose of 100 μg (data not shown), excluding a role for reduced MWCNT mass in the absence of NT3 toxicity. Second, the specific surface area was smaller with the coated MWCNT than with NT1. However, NT2 and NT3 had similar specific surface areas, yet different toxicities. Third, cell internalization was less marked with NT3 than with the other MWCNT. Similarly, Pan and coworkers [38] showed that coating TiO_2 _nanoparticles with a dense grafted polymer brush that exhibited hydrophobic properties, also exhibited by our NT3, prevented adherence to the cell membrane and hence penetration into the cell and induction of oxidative stress. Indeed, cells adhere less well to hydrophobic than to hydrophilic biomaterials [39]. However, the reduced internalization of NT3 was probably not the only mechanism explaining the absence of toxicity of this material, since the *in vivo *experiments showed similar numbers of alveolar macrophages with MWCNT-containing vacuoles in the NT1 and NT3 groups. Fourth, NT3 may have a decreased ability to induce an oxidative and inflammatory response once internalized, compared to the other two MWCNT. This mechanism is consistent with the presence on the NT3 surface of large numbers of ozone oxidizable groups, compared to the other two MWCNT. These oxidizable groups may act as free radical scavengers [40] thereby decreasing the oxidative stress and consecutive inflammation in NT3-exposed cells and animals. Further studies are needed to determine whether such a mechanism is at play.

We administered MWCNT intratracheally to investigate their effects on the lung. We are aware that inhalation should be preferred over intratracheal instillation for toxicological investigations [41] but we believe our study is relevant to the analysis of the importance of surface characteristics as a determinant of MWCNT toxicity, as we compared the effects of the three materials administered to mice in the same way and using the same experimental set-up. Furthermore, the consistency of the results from our simultaneous *in vitro *and *in vivo *investigations supports the validity of our findings.

## Conclusions

In conclusion, to the best of our knowledge, this study supplies the first evidence that using polymer-coating is a useful strategy to diminish adverse effects of CNT. Such tool to modify the surface environment of industrially produced CNT may hold promise for improving occupational safety, as well as general user's safety.

## Competing interests

The authors declare that they have no competing interests.

## Authors' contributions

LT, CB, JB, and SL designed the study. LT, CB and ASD performed the biological assays. AS and MR performed the physicochemical measurements. JB and SL drafted the manuscript, and CB and MR helped with the final version. All authors read and approved the final manuscript.

## Supplementary Material

Additional file 1 **Representative scanning electron microscopy (SEM) of CNT uncoated (NT1) and coated with carboxylic polyacid or polystyrene polybutadiene polymetacrylate of methyl polymers (NT2 and NT3 respectively)**. Click here for file

Additional file 2 **Representative transmission electronic microscopy (TEM) images of RAW 264.7 cells exposed for 24 h to 10 μg/ml of NT1**. **Panel a**: focus is performed on cellular structures. **Panel b**: focus is performed on individual CNT inside the cell. Abbreviations are the same as in Figure 1. Click here for file

Additional file 3 **Cell viability assessed by MTT assay**. Viability was expressed as a percentage of control cell values, after 6h exposure to 100 μg/ml CB, Crocidolite, or 0.1 to 100 μg/ml NT1, NT2 or NT3. Abbreviations are the same as in Figure 1 and 2. Results are represented as box and whiskers for values obtained in 3-6 experiments. *: p < 0.05 *vs *control condition. Click here for file

Additional file 4 **Cell viability assessed by MTT assay**. Viability was expressed as a percentage of control cell values, after 24h exposure to Polymers alone (Carb. Pol.: carboxylic polyacid polymer, PMMA Pol.: polystyrene polybutadiene polymethylmethacrylate polymer), or 100 μg/ml CB, Crocidolite, NT1, NT2 or NT3. Abbreviations are the same as in Figure 1 and 2. Results are represented as box and whiskers for values obtained in 3-6 experiments. *: p < 0.05 *vs *control condition. Click here for file

Additional file 5 **Effect of NT1, NT2 or NT3 in a dose of 100 or 200 μg/mouse on total cell content of bronchoalveolar lavage fluid (BALF) after 1 day exposure**. Results are represented as box and whisker plots of values from 4-6 animals per group. Abbreviations are the same as in Additional file 4. *: *p *< 0.05 compared to control mice. Click here for file

Additional file 6 **Representative optical microscopy images of cells from BAL after 1 day exposure to CNT vehicle or 100 μg/mouse NT1, NT2 or NT3 (magnification ×10)**. Abbreviations are the same as in Figure 1 and 2. Click here for file

Additional file 7 **Lung histology 1 week after a single intratracheal instillation of CNT vehicle or NT1, NT2 or NT3 (100 μg/mouse, magnification ×20)**. Abbreviations are the same as in Figure 1 and 2. Inserts are higher magnification (×40) of CNT agglomerates. Click here for file

Additional file 8 **Lung histology 3 months after a single intratracheal instillation of CNT vehicle or NT1, NT2 or NT3 (100 μg/mouse, magnification ×20)**. Abbreviations are the same as in Figure 1 and 2. Inserts are higher magnification (×40) of CNT agglomerates. Click here for file

Additional file 9 **Lung histology 6 months after a single intratracheal instillation of CNT vehicle or NT1, NT2 or NT3 (100 μg/mouse, magnification ×20)**. Abbreviations are the same as in Figure 1 and 2. Inserts are higher magnification (×40) of CNT agglomerates. Click here for file

Additional file 10 **Higher magnification (×40) of clusters of cells surrounding visible NT1 (left panel) or NT2 (right panel) agglomerates, 1 month post-instillation**. Click here for file
