# Supplementary figures and images for: Coating carbon nanotubes with a polystyrene-based polymer protects against pulmonary toxicity
Source: Part Fibre Toxicol. 2011 Jan 21;8:3. doi: 10.1186/1743-8977-8-3 (PMC3030506; doi:10.1186/1743-8977-8-3)

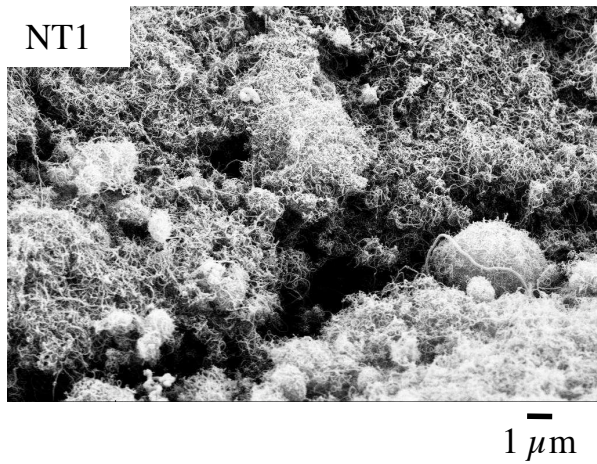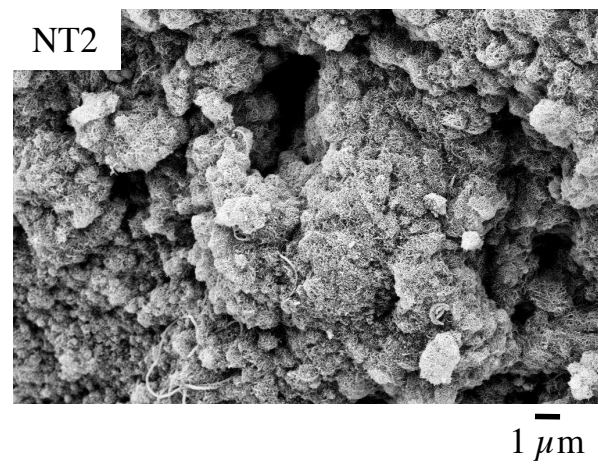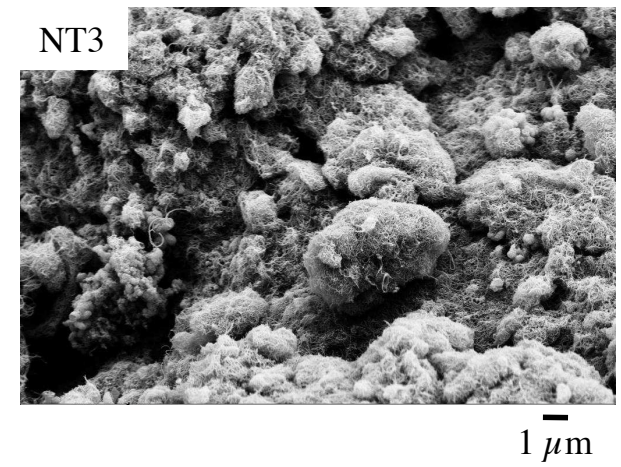

Supplement: Additional file 1 — Representative scanning electron microscopy (SEM) of CNT uncoated (NT1) and coated with carboxylic polyacid or polystyrene polybutadiene polymetacrylate of methyl polymers (NT2 and NT3 respectively). [file 1743-8977-8-3-S1.PDF]

Figure E2

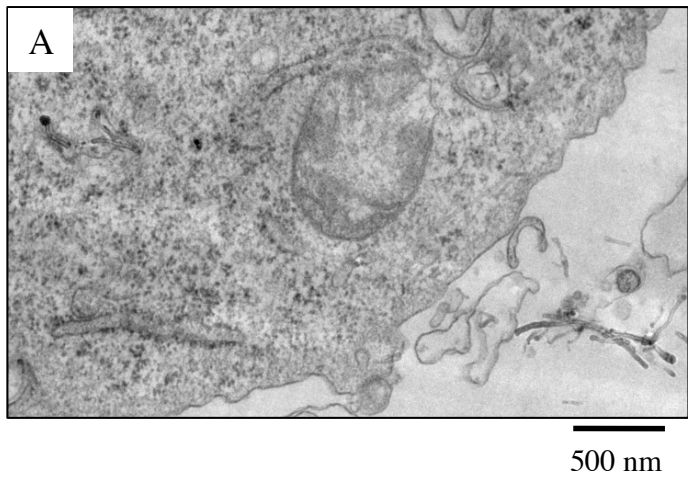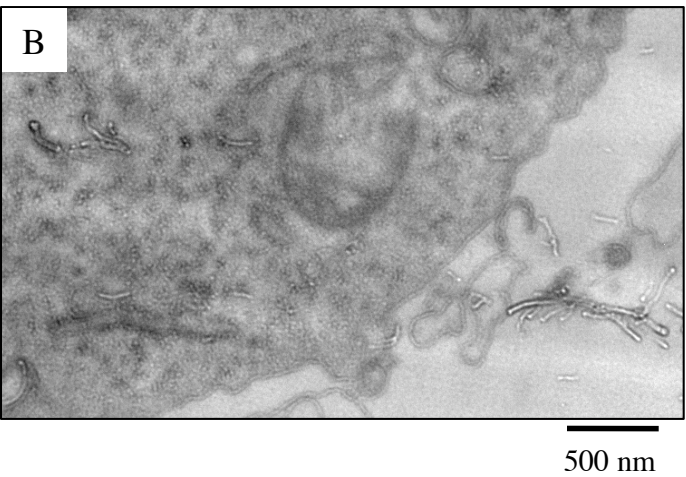

Supplement: Additional file 2 — Representative transmission electronic microscopy (TEM) images of RAW 264.7 cells exposed for 24 h to 10 μg/ml of NT1. Panel a: focus is performed on cellular structures. Panel b: focus is performed on individual CNT inside the cell. Abbreviations are the same as in Figure 1. [file 1743-8977-8-3-S2.PDF]

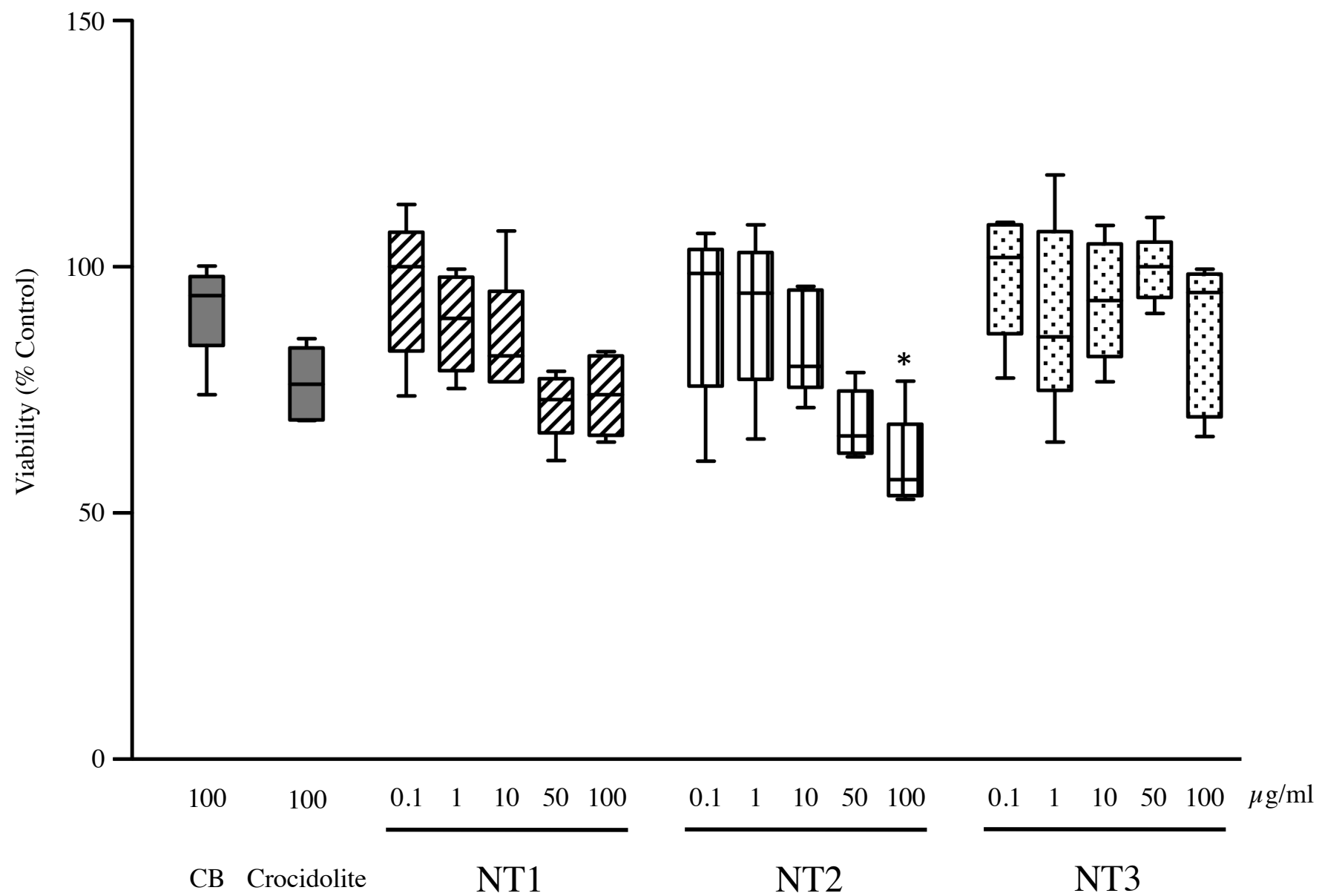

Supplement: Additional file 3 — Cell viability assessed by MTT assay. Viability was expressed as a percentage of control cell values, after 6h exposure to 100 μg/ml CB, Crocidolite, or 0.1 to 100 μg/ml NT1, NT2 or NT3. Abbreviations are the same as in Figure 1 and 2. Results are represented as box and whiskers for values obtained in 3-6 experiments. *: p < 0.05 vs control condition. [file 1743-8977-8-3-S3.PDF]

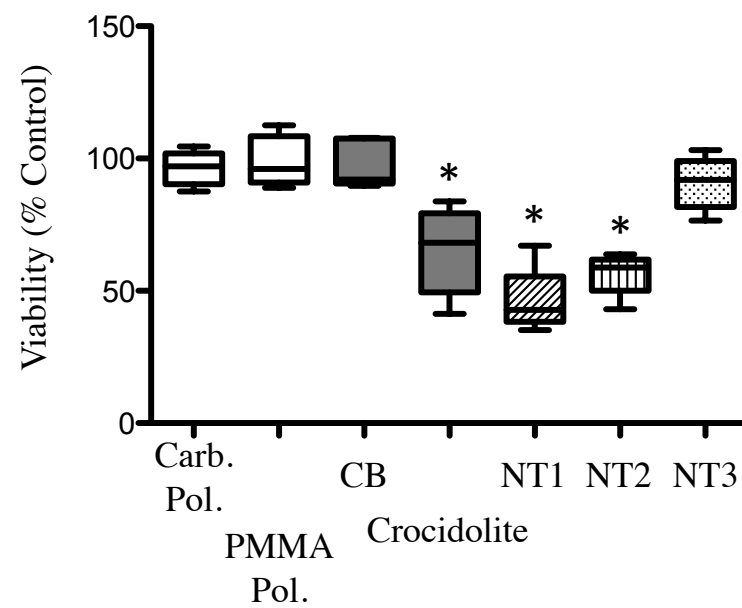

Supplement: Additional file 4 — Cell viability assessed by MTT assay. Viability was expressed as a percentage of control cell values, after 24h exposure to Polymers alone (Carb. Pol.: carboxylic polyacid polymer, PMMA Pol.: polystyrene polybutadiene polymethylmethacrylate polymer), or 100 μg/ml CB, Crocidolite, NT1, NT2 or NT3. Abbreviations are the same as in Figure 1 and 2. Results are represented as box and whiskers for values obtained in 3-6 experiments. *: p < 0.05 vs control condition. [file 1743-8977-8-3-S4.PDF]

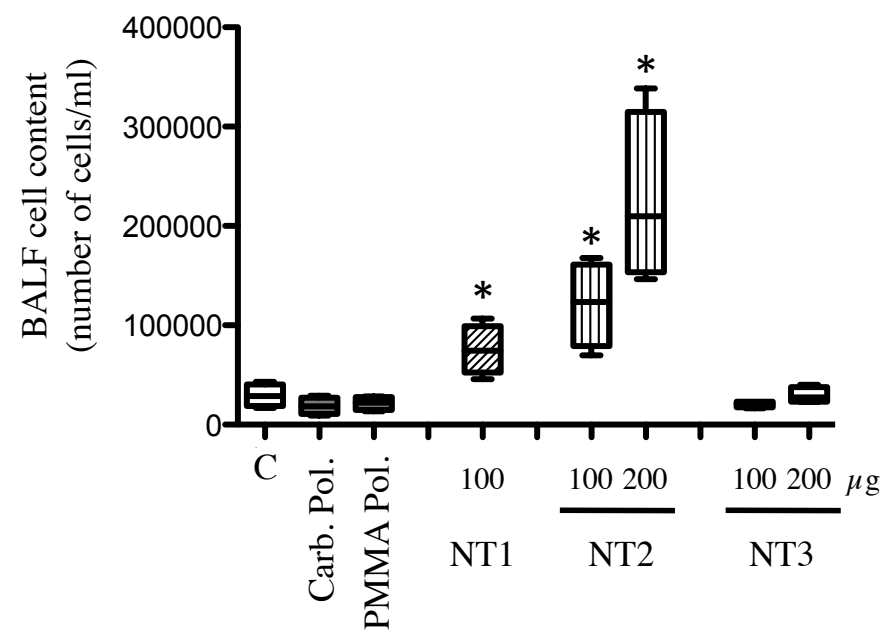

Supplement: Additional file 5 — Effect of NT1, NT2 or NT3 in a dose of 100 or 200 μg/mouse on total cell content of bronchoalveolar lavage fluid (BALF) after 1 day exposure. Results are represented as box and whisker plots of values from 4-6 animals per group. Abbreviations are the same as in Additional file 4. *: p < 0.05 compared to control mice. [file 1743-8977-8-3-S5.PDF]

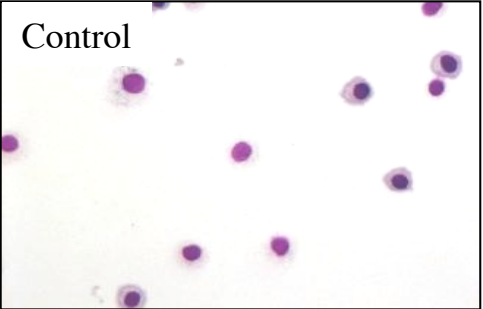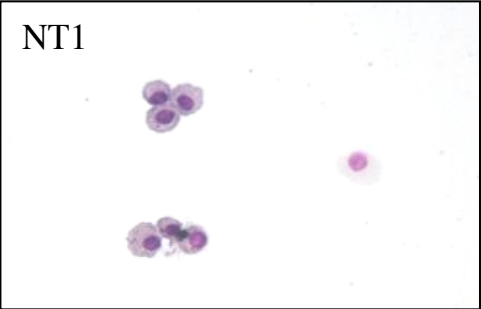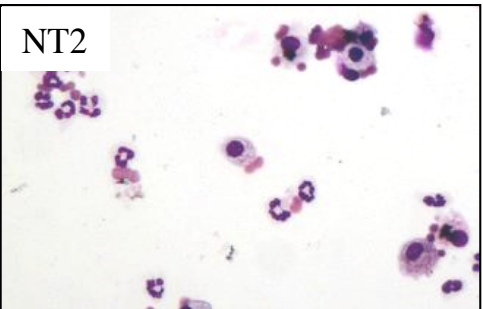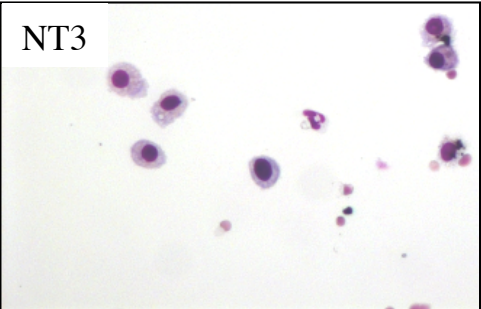

Supplement: Additional file 6 — Representative optical microscopy images of cells from BAL after 1 day exposure to CNT vehicle or 100 μg/mouse NT1, NT2 or NT3 (magnification ×10). Abbreviations are the same as in Figure 1 and 2. [file 1743-8977-8-3-S6.PDF]

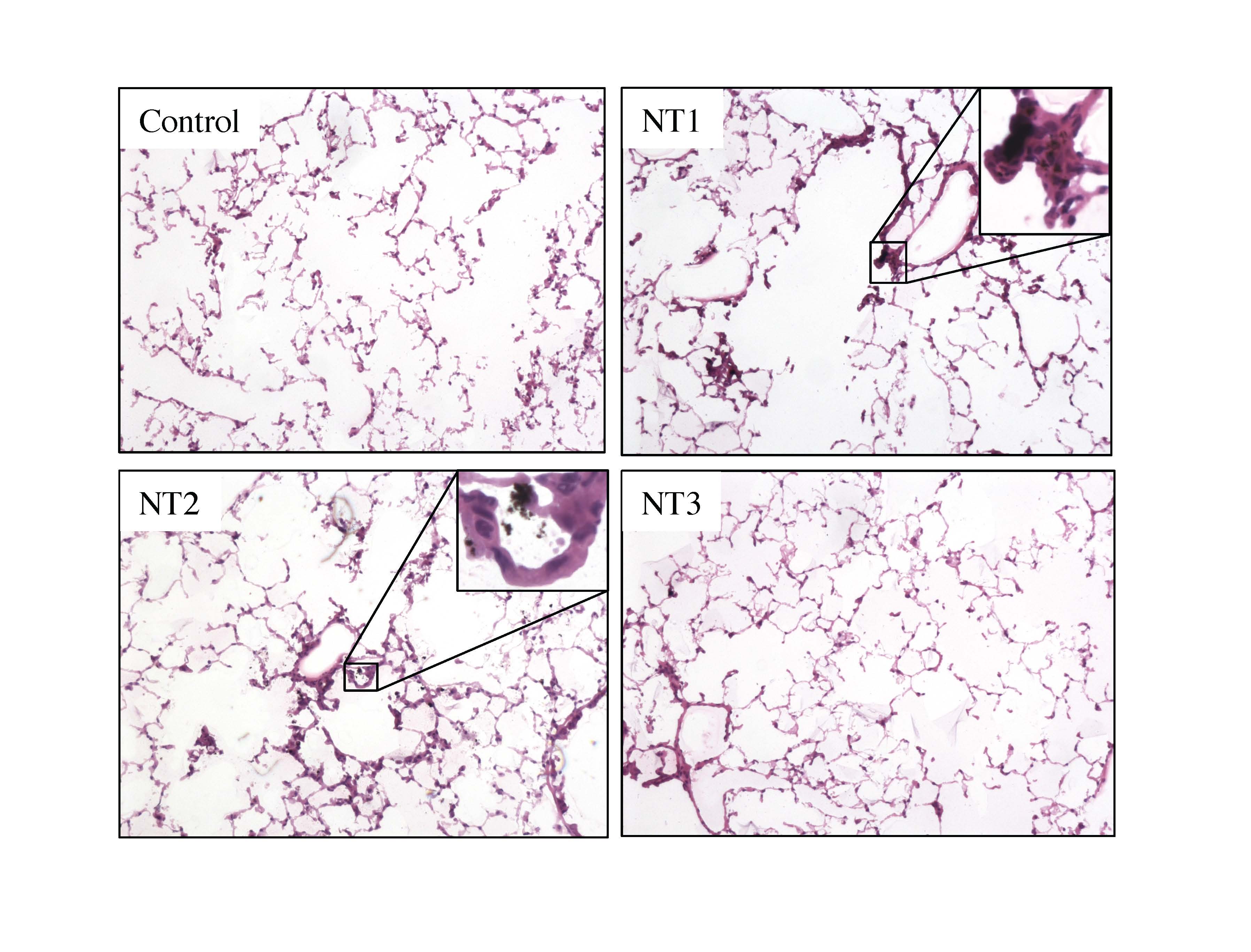

Supplement: Additional file 7 — Lung histology 1 week after a single intratracheal instillation of CNT vehicle or NT1, NT2 or NT3 (100 μg/mouse, magnification ×20). Abbreviations are the same as in Figure 1 and 2. Inserts are higher magnification (×40) of CNT agglomerates. [file 1743-8977-8-3-S7.JPEG]

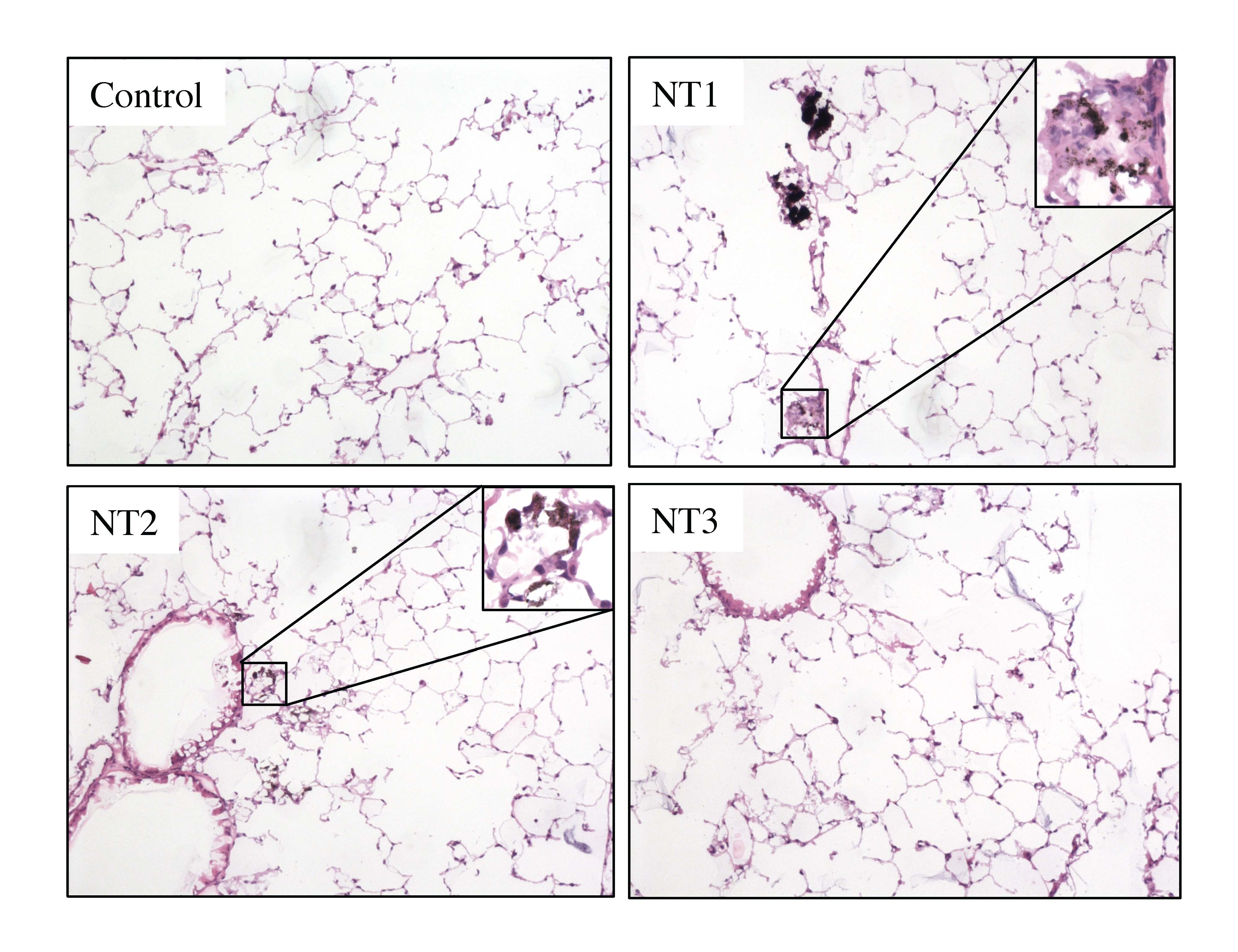

Supplement: Additional file 8 — Lung histology 3 months after a single intratracheal instillation of CNT vehicle or NT1, NT2 or NT3 (100 μg/mouse, magnification ×20). Abbreviations are the same as in Figure 1 and 2. Inserts are higher magnification (×40) of CNT agglomerates. [file 1743-8977-8-3-S8.JPEG]

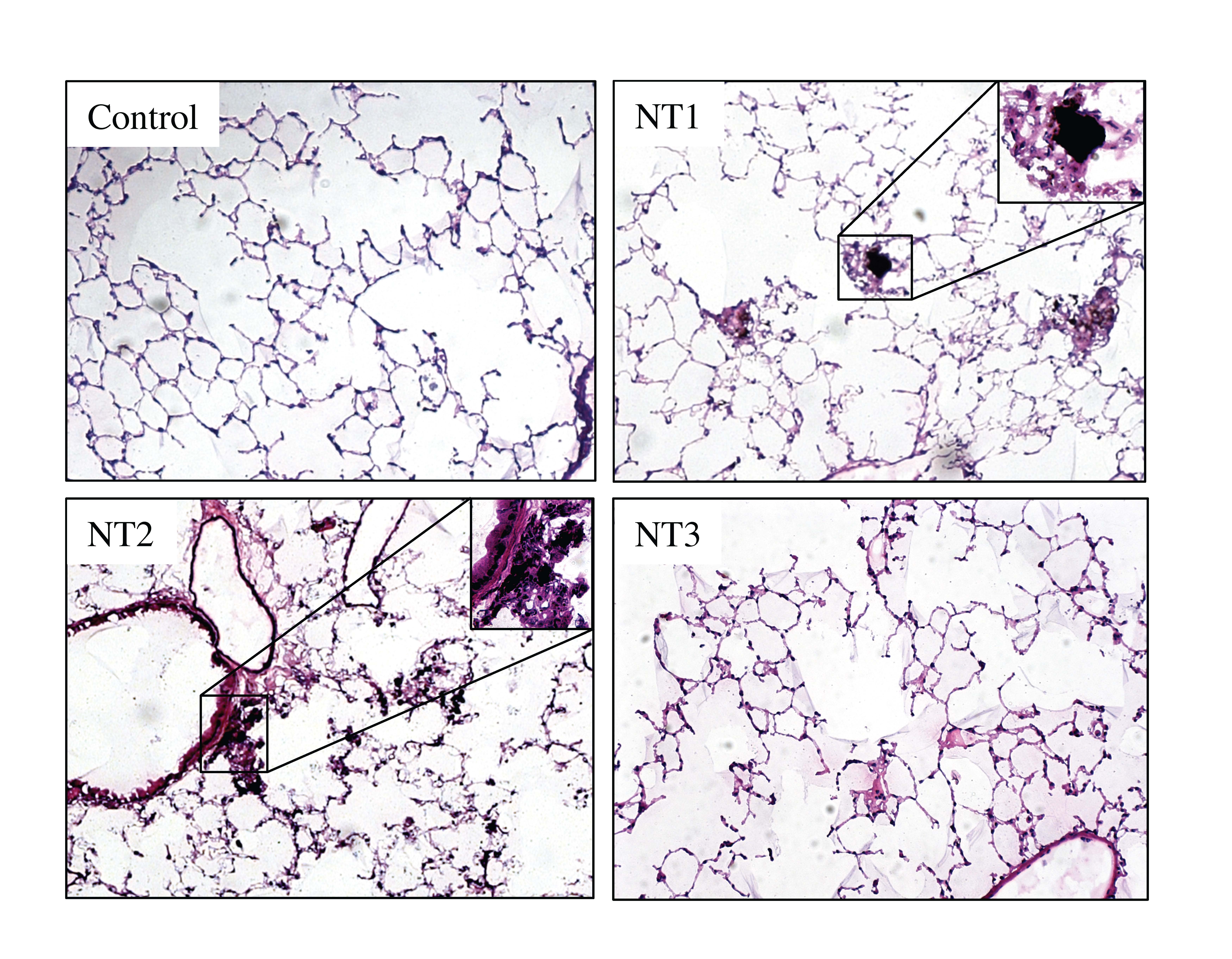

Supplement: Additional file 9 — Lung histology 6 months after a single intratracheal instillation of CNT vehicle or NT1, NT2 or NT3 (100 μg/mouse, magnification ×20). Abbreviations are the same as in Figure 1 and 2. Inserts are higher magnification (×40) of CNT agglomerates. [file 1743-8977-8-3-S9.JPEG]

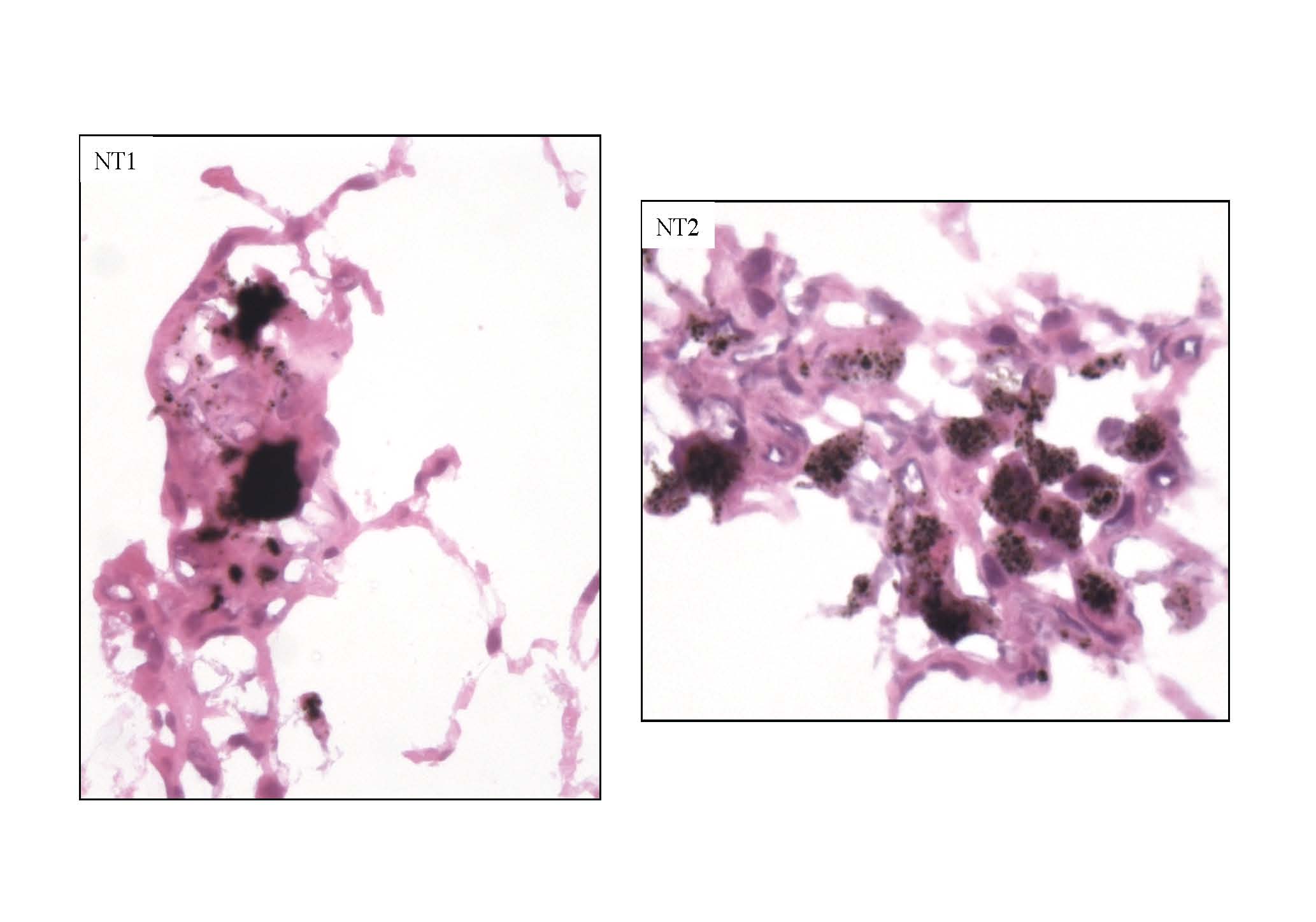

Supplement: Additional file 10 — Higher magnification (×40) of clusters of cells surrounding visible NT1 (left panel) or NT2 (right panel) agglomerates, 1 month post-instillation. [file 1743-8977-8-3-S10.JPEG]
